# The accuracy of fully-automated algorithms for the surveillance of central venous catheter-related bloodstream infection in hospitalised patients

**DOI:** 10.1186/s13756-024-01373-w

**Published:** 2024-02-05

**Authors:** Moa Karmefors Idvall, Hideyuki Tanushi, Andreas Berge, Pontus Nauclér, Suzanne Desirée van der Werff

**Affiliations:** 1 Department of Medicine Solna, Division of Infectious Diseases, Karolinska Institutet, 171 77 Stockholm, Sweden; 2 Department of Data Processing and Analysis, Karolinska University Hospital, Stockholm, Sweden; 3 Department of Infectious Diseases, Karolinska University Hospital, Stockholm, Sweden

**Keywords:** Automated surveillance, Algorithms, Catheter-related infection, Central venous catheter-related bloodstream infection, Electronic health record data, Healthcare-associated infections

## Abstract

**Background:**

Continuous surveillance for healthcare-associated infections such as central venous catheter-related bloodstream infections (CVC-BSI) is crucial for prevention. However, traditional surveillance methods are resource-intensive and prone to bias. This study aimed to develop and validate fully-automated surveillance algorithms for CVC-BSI.

**Methods:**

Two algorithms were developed using electronic health record data from 1000 admissions with a positive blood culture (BCx) at Karolinska University Hospital from 2017: (1) Combining microbiological findings in BCx and CVC cultures with BSI symptoms; (2) Only using microbiological findings. These algorithms were validated in 5170 potential CVC-BSI-episodes from all admissions in 2018–2019, and results extrapolated to all potential CVC-BSI-episodes within this period (n = 181,354). The reference standard was manual record review according to ECDC’s definition of microbiologically confirmed CVC-BSI (CRI3-CVC).

**Results:**

In the potential CVC-BSI-episodes, 51 fulfilled ECDC’s definition and the algorithms identified 47 and 49 episodes as CVC-BSI, respectively. Both algorithms performed well in assessing CVC-BSI. Overall, algorithm 2 performed slightly better with in the total period a sensitivity of 0.880 (95%-CI 0.783–0.959), specificity of 1.000 (95%-CI 0.999–1.000), PPV of 0.918 (95%-CI 0.833–0.981) and NPV of 1.000 (95%-CI 0.999–1.000). Incidence according to the reference and algorithm 2 was 0.33 and 0.31 per 1000 in-patient hospital-days, respectively.

**Conclusions:**

Both fully-automated surveillance algorithms for CVC-BSI performed well and could effectively replace manual surveillance. The simpler algorithm, using only microbiology data, is suitable when BCx testing adheres to recommendations, otherwise the algorithm using symptom data might be required. Further validation in other settings is necessary to assess the algorithms’ generalisability.

## Background

Healthcare-associated infections (HAIs) are common adverse events, affecting millions of patients annually, imposing a significant burden on the healthcare system and leading to extended hospital stays, increased morbidity, mortality, and higher costs [1–3]. Bloodstream infections (BSIs) constitute nearly 11% of all HAIs, affecting around 375,000 patients yearly in Europe [3]. Among these, central venous catheter-related BSIs (CVC-BSIs), associated with the use of these medical devices, remain a significant concern despite recent progress in their prevention [4, 5]. The incidence of CVC-BSI is often considered a key indicator of the effectiveness of infection prevention and control measures in healthcare settings [5].

A substantial proportion of HAIs are preventable [1, 4, 5]. Continuous surveillance with feedback to healthcare personnel and stakeholders is essential to effectively allocate resources and evaluate interventions [6, 7]. Still, most HAI surveillance relies on time-consuming and resource-intensive manual record review, prone to subjective interpretation and surveillance bias [8–10]. However, with the adoption of electronic health record (EHR) systems, detailed digital EHR data is accessible which is enabling the development of automated surveillance methods, thereby reducing workload, and providing standardised and continuous surveillance data [11, 12]. Nonetheless, these surveillance algorithms must undergo thorough validation before implementation.

Automated surveillance algorithms for CVC-BSI have been developed [13–26]. However, to our knowledge, the majority were based on an ICU population [13–19], were semi-automated [13–16, 20–23], and nearly all used the central line-associated BSI (CLABSI) criteria of the Centers for Disease Control and Prevention (CDC) as reference [13–15, 17–26] and none the microbiologically confirmed CVC-BSI (CRI3-CVC) definition of the European Centre for Disease Prevention and Control (ECDC).

In this study, the aim was to develop fully-automated rule-based surveillance algorithms using EHR data for the detection of CVC-BSI in hospitalised patients, and validate it against manual record review according to ECDC’s CRI3-CVC definition. The best performing algorithm was applied over a four-year period to visualise the incidence of CVC-BSI.

## Methods

### Study design and data source

**Fig. 1:**
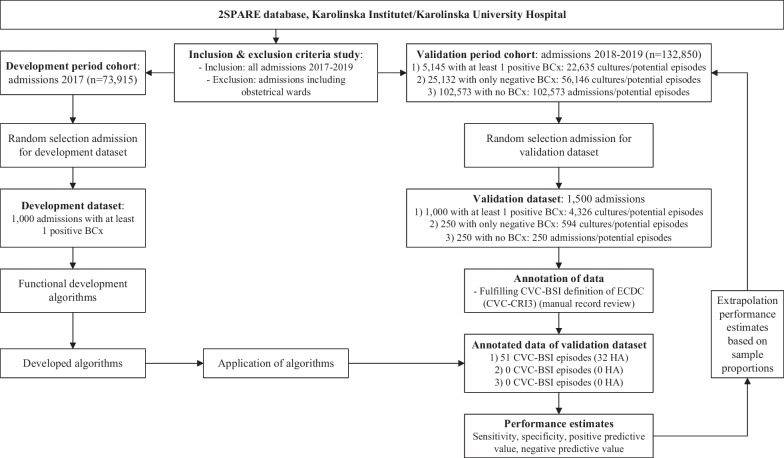
Flow chart of study. *BCx*: blood culture; *CRI3-CVC*: microbiologically confirmed central venous catheter-related bloodstream infection; *CVC-BSI:* central venous catheter-related bloodstream infection; *ECDC:* European Centre for Disease Prevention and Control; *HA:* healthcare-associated. *Positive BCx:* BCx with any growth of microorganisms (pathogens and/or contaminants). *CVC-BSI-episode*: all BCx taken during admission were regarded as potential CVC-BSI-episodes, and each admission without BCx taken counted as one potential CVC-BSI-episode

This retrospective cohort study used prospectively entered EHR data from the Karolinska University Hospital (KUH) stored in the 2SPARE (2020 started Stockholm/Sweden Proactive Adverse Events REsearch) database. KUH is a tertiary care academic centre in Stockholm, Sweden with 1100 beds divided between two sites (Huddinge and Solna), which serves a population of 2.3 million inhabitants, i.e., the entire population of Region Stockholm. Data included demographics, hospital administrative data, disease and procedure codes, microbiological results, physiological parameters, medication, and medical notes. For this study hospital admissions between 2017 and 2019 were used (Fig. 1). Admissions which included obstetric wards were excluded due to lack of complete data. For the intensive care unit (ICU) no structured data on physiological parameters were available.

The algorithms were functionally developed in a simple random sample of 1000 admissions with a positive blood culture (BCx) from 2017. Only (semi-)structured variables were used. Admissions from 2018–2019 (n = 132,850) were used as the validation period cohort (Fig. 1). These admissions were divided into three groups: (1) admissions with at least one positive BCx (n = 5145), (2) admissions with only negative BCx (n = 25,132), and (3) admissions without BCx performed (n = 102,573). From these three groups 1000, 250 and 250 admissions, respectively, were selected via simple random selection for the validation dataset. In this validation dataset the presence of CVC-BSI was assessed by three trained professionals by manual record review as gold standard. The reviewers were blinded for the algorithm results and forty cases were reviewed in overlap resulting in almost perfect agreement (95.8–100%) between them, with a Cohen’s kappa of 0.90–1.00. Complicated cases were discussed and classified using a consensus decision by all reviewers. All BCx during admission were regarded as potential CVC-BSI-episodes, and an admission with no BCx counted as one potential CVC-BSI-episode. The study was approved by the Regional Ethical Review Board in Stockholm under permission no. 2018/1030-31.

### Case definition and reference standard of CVC-BSI

The assessment of CVC-BSI during manual record review was performed according to ECDC’s BSI and CRI3-CVC definitions at the time of this study [27]. Contaminant was defined according to the common commensal list of CDC [28]. All CVCs fulfilling the criteria of ECDC and CDC were assessed [27, 29]. All CVC-BSI were recorded, but the healthcare-associated (HA) classification was based on ECDC’s definition [27].

### Algorithms

**Fig. 2:**
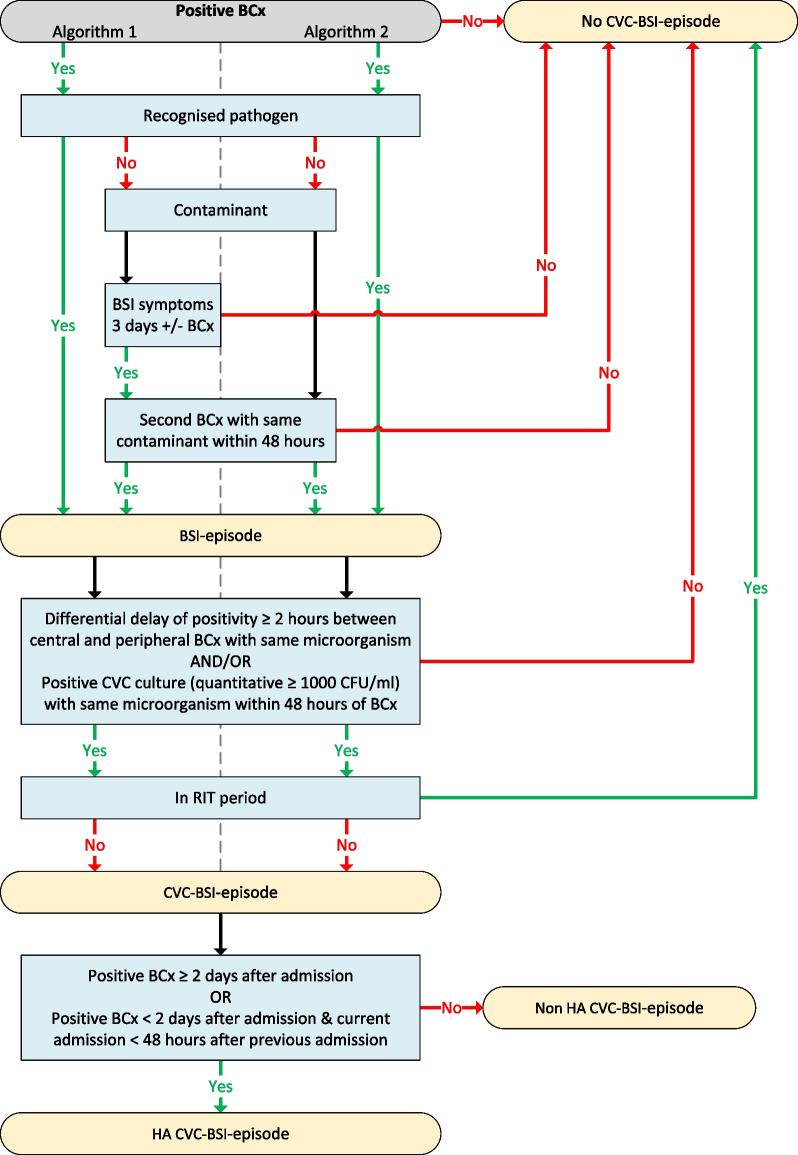
Flow diagram of the two rule-based surveillance algorithms for central venous catheter-related bloodstream infection. *BCx:* blood culture; *BSI:* bloodstream infection; *CFU:* colony-forming unit; *CVC:* central venous catheter; *HA:* healthcare-associated. *Positive BCx:* any BCx with growth of microorganisms (pathogens and/or contaminants). *BSI symptoms*: fever (> 38 °C) and/or hypotension (systolic blood pressure < 90 mmHg or diastolic blood pressure ≤ 60 mmHg). *CVC-BSI-episode*: all BCx taken during admission were regarded as potential CVC-BSI-episodes, and each admission without BCx taken counted as one potential CVC-BSI-episode. *RIT*: repeat infection timeframe, i.e., within 14 days after the first positive BCx of a previous CVC-BSI-episode

Two rule-based algorithms for CVC-BSI detection were developed in the development dataset (Fig. 2):

1. Relevant microbiological findings in BCx and CVC cultures combined with the presence of BSI symptoms.
2. Only relevant microbiological findings in BCx and CVC cultures.

For BSI, a positive BCx was defined as any growth of microorganisms and the division between a recognised pathogen or contaminant was based on the organism classification list of the CDC [28]. For recognised pathogens one BCx was sufficient. For contaminants two different BCx taken within 48 h of each other should be present together with the presence of BSI symptoms. BSI symptoms comprised fever (> 38 °C) and hypotension, the latter defined as a systolic blood pressure < 90 mmHg or a diastolic blood pressure ≤ 60 mmHg [29]. BSI symptoms had to be present within three days before or after the BCx based on CDC’s infection window period [30]. To define a BSI as CVC-related either (1) a positive CVC-tip culture (quantitative ≥ 10^3^ CFU/ml) with the same microorganism (species level) taken within 48 h after the BCx or (2) a peripheral and central BCx, taken max. 15 min apart from each other, with the same microorganism and ≥ 2 h differential time to positivity between both BCx (central BCx < peripheral BCx) had to be present. These two criteria also served as proxy for the BSI occurring before or after catheter removal. CDC’s 14-day repeat infection timeframe (RIT) criterion was used to assess when another CVC-BSI-episode could be registered when multiple CVC-BSI-episodes where present during one admission [30]. All CVC-BSI were assessed, but additionally they were defined as HA when the BCx was taken two days or more after admission (on day three or later where day of admission is day one) or within two days after admission (on day one or two of admission) if a previous admission within 48 h was present.

### Statistical analysis

Data acquisition, management and analysis were performed using R (version 4.1.0) and Python (version 3.7), and in accordance with current regulations concerning privacy and ethics. Continuous variables are presented as median with interquartile ranges (IQR) and categorical variables as numbers with percentages. For algorithm performance, the sensitivity, specificity, positive predictive value (PPV), negative predictive value (NPV), area under the receiver operating characteristic curve (AUROC), and kappa statistics were assessed. The 95% confidence interval (95%CI) for estimates in the validation dataset were calculated using the asymptotic variance with Wilson score method. Results from the validation dataset were extrapolated to the validation period cohort of 2018–2019 to obtain performance estimates of the algorithms in the complete target population (Fig. 1). The CI for these estimates were calculated as the 2.5th and 97.5th percentiles of point estimates obtained from 10,000 bootstrap samples for each of the three groups. To account for uncertainty, the bootstrapping was performed before extrapolating the proportions from the validation dataset to the validation period cohort [31]. Finally, the best performing algorithm was applied to data of 2017–2020 to visualise the incidence of CVC-BSI per 1000 in-patient hospital-days by continuous automated surveillance.

## Results

In the validation dataset 4326 BCx, of which 1766 positive (40.8%), were present among the 1000 admissions with at least one positive BCx, and 594 BCx in the 250 admissions with only negative BCx. Together with the 250 admissions with no BCx taken, this amounted to 5170 potential CVC-BSI-episodes used for the calculations of algorithm performance (Table 1 and Fig. 1). There were 181,354 potential CVC-BSI-episodes during admissions of the validation period cohort of 2018–2019 (n = 132,850) used for the calculation of extrapolated algorithm performance (Fig. 1). Of the 78,781 BCx present during this period, 9340 were positive (11.9%).

**Table 1:** Characteristics of three admission groups in validation dataset

Characteristics	All	Admissions with positive BCx	Admissions with negative BCx	Admissions with no BCx
Potential CVC-BSI episodes, n	5170	4326	594	250
BCx, n	4920	4326	594	0
Admissions, n	1500	1000	250	250
Patients, n	1470	972	248	250
Sex, n (% of patients)				
Female	675 (45.9)	430 (44.2)	122 (49.2)	123 (49.2)
Male	792 (53.9)	541 (55.7)	126 (50.8)	125 (50.0)
Unknown	3 (0.2)	1 (0.1)	0 (0)	2 (0.8)
Age (years), median [IQR] of admissions	64 [41–75]	67 [48–75]	60 [29–75]	54 [30–69]
Length of stay (days), median [IQR] of admissions	8 [4–16]	10 [16–21]	7 [4–14]	3 [2–6]
ICU admission, n (% of admissions)	198 (13.2)	159 (15.9)	33 (13.2)	6 (2.4)
In-hospital mortality, n (% of admissions)	100 (6.7)	91 (9.1)	7 (2.8)	2 (0.8)
ECDC CVC-BSI, n (% of episodes)	51 (1.0)	51 (1.2)	0 (0.0)	0 (0.0)
ECDC HA CVC-BSI, n (% of episodes)	32 (0.6)	32 (0.7)	0 (0.0)	0 (0.0)

*BCx:* blood culture; *CVC-BSI:* central venous catheter-related bloodstream infection; *ECDC:* European Centre for Disease Prevention and Control; *HA:* healthcare-associated; *ICU:* intensive care unit; *IQR:* interquartile range

Overall, the patients within the validation dataset with positive BCx were older and consisted of more males than the patients with negative or no BCx during admission (Table 1). The length of hospital stay, number of ICU admissions and in-hospital mortality was highest in admissions with positive BCx and lowest in admissions with no BCx.

### Algorithms performance

No CVC-BSI-episodes were identified during manual record review in admissions with only negative BCx or no BCx. In the 4326 potential CVC-BSI-episodes of admissions with at least one positive BCx, 51 episodes (1.2%) fulfilled ECDC’s CRI3-CVC definition, and 32 episodes (0.7%) the criteria for HA (Table 1). The two algorithms classified 47 (1.09%) and 49 (1.13%) as CVC-BSI-episodes, respectively, and 26 (0.60%) and 27 (0.62%) as HA, respectively.

In the validation dataset with positive BCx as well as the total validation period cohort (extrapolated data) for all CVC-BSI, the algorithm based on relevant microbiological findings in BCx and CVC cultures combined with the presence of BSI symptoms (algorithm 1) had a high sensitivity and PPV to determine CVC-BSI according to ECDC’s definition with a sensitivity of 0.861 (95%CI 0.764–0.959) and PPV of 0.935 (95%CI 0.850–1.000) in the validation period cohort (Table 2). The algorithm based on only relevant microbiological findings in BCx and CVC cultures (algorithm 2) had a sensitivity of 0.880 (95%CI 0.783–0.959) and PPV of 0.918 (95%CI 0.833–0.981) in the validation period cohort. The specificity and NPV was high (Table 2).

**Table 2:** Performance characteristics two rule-based algorithms for central venous catheter-related bloodstream infection according to ECDC definition

All CVC-BSI	TP	FP	FN	TN	Sensitivity (95%CI)	Specificity (95%CI)	PPV (95%CI)	NPV (95%CI)	AUROC (95%CI)	Kappa (95%CI)
Validation dataset with admissions with at least one positive BCx (potential CVC-BSI-episodes^a^ n = 4326)										
Algorithm 1^b^	44	3	7	4272	0.863 (0.743–0.932)	0.999 (0.998–1.000)	0.936 (0.828–0.978)	0.998 (0.997–0.999)	0.931 (0.883–0.979)	0.897 (0.867–0.927)
Algorithm 2^c^	45	4	6	4271	0.882 (0.766–0.945)	0.999 (0.998–1.000)	0.918 (0.808–0.968)	0.999 (0.997–0.999)	0.941 (0.896–0.985)	0.899 (0.869–0.929)
Extrapolated results^d^ for validation period cohort (potential CVC-BSI-episodes^a^ n = 181,354)										
Algorithm 1^b^	–	–	–	–	0.861 (0.764–0.959)	1.000 (0.999–1.000)	0.935 (0.850–1.000)	1.000 (0.999–1.000)	0.931 (0.910–0.951)	0.897 (0.892–0.901)
Algorithm 2^c^	–	–	–	–	0.880 (0.783–0.959)	1.000 (0.999–1.000)	0.918 (0.833–0.981)	1.000 (0.999–1.000)	0.940 (0.921–0.960)	0.899 (0.894–0.903)

HA CVC-BSI	TP	FP	FN	TN	Sensitivity (95%CI)	Specificity (95%CI)	PPV (95%CI)	NPV (95%CI)	AUROC (95%CI)	Kappa (95%CI)
Validation dataset with admissions with at least one positive BCx (potential CVC-BSI-episodes^a^ n = 4326)										
Algorithm 1^b^	25	1	7	4293	0.781 (0.612–0.890)	1.000 (0.999–1.000)	0.962 (0.811–0.993)	0.998 (0.997–0.999)	0.891 (0.818–0.963)	0.861 (0.832–0.891)
Algorithm 2^c^	26	1	6	4293	0.813 (0.647–0.911)	1.000 (0.999–1.000)	0.963 (0.817–0.993)	0.999 (0.997–0.999)	0.906 (0.837–0.975)	0.881 (0.851–0.910)
Extrapolated results^d^ for validation period cohort (potential CVC-BSI-episodes^a^ n = 181,354)										
Algorithm 1^b^	–	–	–	–	0.784 (0.629–0.910)	1.000 (0.999–1.000)	0.956 (0.868–1.000)	1.000 (0.999–1.000)	0.892 (0.861–0.924)	0.861 (0.857–0.866)
Algorithm 2^c^	–	–	–	–	0.814 (0.659–0.940)	1.000 (0.999–1.000)	0.958 (0.873–1.000)	1.000 (0.999–1.000)	0.907 (0.878–0.937)	0.880 (0.876–0.885)

*AUROC:* area under the receiver operating characteristic curve; *BCx:* blood culture; *CVC-BSI:* central venous catheter-related bloodstream infection; *ECDC:* European Centre for Disease Prevention and Control; *FN:* false negative; *FP:* false positive; *HA:* healthcare-associated; *NPV:* negative predictive value; *PPV:* positive predictive value; *TN:* true negative; *TP:* true positive

^a^*CVC-BSI-episode*: all performed BCx were regarded as potential CVC-BSI-episodes during an admission, and admissions with no BCx counted as one potential CVC-BSI-episode

^b^*Algorithm 1*: Relevant microbiological findings in BCx and CVC cultures combined with the presence of BSI symptoms

^c^*Algorithm 2*: Only relevant microbiological findings in BCx and CVC cultures

^d^The extrapolated results of the algorithms from the validation dataset to the validation period cohort (2018–2019) were based on the sampling proportion of potential CVC-BSI-episodes from the three different groups: 1) admissions with at least one positive BCx; 2) admissions with only negative BCx and; 3) admissions without a BCx

In the HA CVC-BSI cases, for both algorithms the sensitivity was lower and PPV higher compared to all CVC-BSI cases (Table 2). In the validation period cohort, the sensitivity was 0.784 (95%CI 0.629–0.910) and the PVV was 0.956 (95%CI 0.868–1.000) for algorithm 1. For algorithm 2 the sensitivity was 0.814 (95%CI 0.659–0.940) and PPV was 0.958 (95%CI 0.873–1.000). Overall, the specificity and NPV was high and similar to those for all CVC-BSI cases (Table 2).

The false negative and false positive cases were mostly related to cases wrongly classified by the annotators or algorithm rules missing for specific clinical situations not present in the development dataset (Table 3). When correcting the misclassification in the reference standard, for algorithm 2 in the validation period cohort the sensitivity would increase to 0.925 (95%CI 0.816–1.000) and 0.939 (95%CI 0.859–1.000) for HA and all CVC-BSI cases, respectively. Theoretically, when also improving algorithm rules, it could even increase to 0.959 (95%CI 0.891–1.000) and 1.000 (95%CI 0.999–1.000) for HA and all CVC-BSI cases, respectively.

**Table 3:** Discrepancy analyses between algorithm results and annotation results (reference), for false-negative and false-positive cases

	All CVC-BSI cases		HA CVC-BSI cases	
	Algorithm 1^a^	Algorithm 2^b^	Algorithm 1^a^	Algorithm 2^b^
Reasons false-negative cases				
True negative case (reference incorrect)	3	3	4	4
Relevant information stored as free text	1	–	2	1
Algorithm rules missing/not matching clinical situation	3	3	1	1
Total number of false-negative cases	7	6	7	6
Reasons false-positive cases				
True positive cases (reference incorrect)	2	2	–	–
No symptoms present (contaminant BCx)	–	1	–	–
Algorithm rules missing/not matching clinical situation	1	1	1	1
Total number of false-positive cases	3	4	1	1

*BCx:* blood culture; *CVC-BSI:* central venous catheter-related bloodstream infection; *HA:* healthcare-associated

^a^*Algorithm 1*: Relevant microbiological findings in BCx and CVC cultures combined with the presence of BSI symptoms

^b^*Algorithm 2*: Only relevant microbiological findings in BCx and CVC cultures

### CVC-BSI rates

The CVC-BSI rate was expressed against several possible denominators for the reference, i.e. manual record review, and the two algorithm results (Table 4). Both algorithms give slightly lower rates compared to the reference with algorithm 2 giving rates closest to the reference.

**Table 4:** Rates central venous catheter-related bloodstream infection based on annotation (reference) and algorithm results

	Validation dataset with admissions with at least one positive BCx					
	All CVC-BSI cases			HA CVC-BSI cases		
	Reference^a^ (n = 51)	Algorithm 1^b^ (n = 47)	Algorithm 2^c^ (n = 49)	Reference^a^ (n = 32)	Algorithm 1^b^ (n = 26)	Algorithm 2^c^ (n = 27)
Potential episodes^d^ (n = 4326)	1.18%	1.09%	1.13%	0.74%	0.60%	0.62%
Positive BCx (n = 1766)	2.89%	2.66%	2.77%	1.81%	1.47%	1.53%
Admissions (n = 1000)	5.1%	4.7%	4.9%	3.2%	2.6%	2.7%
1000 in-patient hospital-days (n = 17,782)	2.87	2.64	2.76	1.80	1.46	1.52

	Extrapolated results to validation period cohort					
	All CVC-BSI cases			HA CVC-BSI cases		
	Reference^a^ (n = 267)	Algorithm 1^b^ (n = 246)	Algorithm 2^c^ (n = 256)	Reference^a^ (n = 167)	Algorithm 1^b^ (n = 137)	Algorithm 2^c^ (n = 142)
Potential episodes^d^ (n = 181,354)	0.15%	0.14%	0.14%	0.09%	0.08%	0.08%
Admissions (n = 132,850)	0.20%	0.19%	0.19%	0.13%	0.10%	0.11%
1000 in-patient hospital-days (n = 817,058)	0.33	0.30	0.31	0.20	0.17	0.17

*BCx:* blood culture; *CVC-BSI:* central venous catheter-related bloodstream infection; *HA:* healthcare-associated

^a^*Reference*: True rate based on manual record review in validation dataset and expected rate based on extrapolation in validation period cohort

^b^*Algorithm 1*: Relevant microbiological findings in BCx and CVC cultures combined with the presence of BSI symptoms

^c^*Algorithm 2*: Only relevant microbiological findings in BCx and CVC cultures

^d^*CVC-BSI-episode*: all performed BCx were regarded as potential CVC-BSI-episodes during an admission, and admissions with no BCx counted as one potential CVC-BSI-episode

**Fig. 3:**
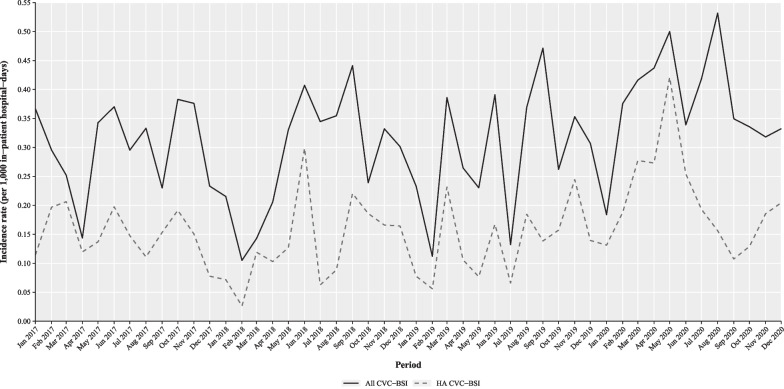
Incidence rate of central venous catheter-related bloodstream infection per 1000 in-patient hospital-days during the period 2017–2020. *BCx:* blood culture; *CVC-BSI:* central venous catheter-related bloodstream infection; *HA:* healthcare-associated. Incidence rate based on application of algorithm which only used relevant microbiological findings in BCx and CVC cultures

Algorithm 2 was applied over the period 2017–2020 to show the incidence of CVC-BSI over time with automated surveillance (Fig. 3). The hospital-wide incidence rate was overall 0.31 per 1000 in-patient hospital-days and ranged from 0.11 to 0.53 for all CVC-BSI, and it was overall 0.16 per 1000 in-patient hospital-days with a range of 0.03 to 0.42 for HA CVC-BSI. The incidence seemed to show a seasonal trend. In 2020, during the COVID-19 pandemic, the incidence increased and during some period the HA CVC-BSI rate seemed to diverge from the overall CVC-BSI rate.

## Discussion

We developed two fully-automated surveillance algorithms for the detection of CVC-BSI based on ECDC’s CRI3-CVC criteria. Both had a comparable and high performance for detecting CVC-BSI. To our knowledge, these are the first fully-automated surveillance algorithms developed for CVC-BSI based on ECDC’s definition.

Both algorithms had good performance and high agreement with the reference standard even though certain criteria of the surveillance definition were not part of the algorithm rules. Checking for pus cultures from the insertion site was excluded since it was assessed to be less common and more difficult to automate. Chills as symptom was excluded because integration would require free-text analysis. Also, no explicit check for the presence of a CVC was done as adequate structured data on insertion and extraction was lacking and it was assumed that the criteria for CVC culture and differential positivity between central and peripheral BCx could serve as proxy. These non-incorporated criteria of the definition were not relevant for the algorithm performance as none of the discrepant cases were related to these criteria. These cases were mostly related to misclassification of CVC-BSI cases during annotation or rules missing in the algorithm due to situations not present in the development data. As reported, when correcting the misclassification in the reference standard and additionally also improving the algorithm rules, the performance of the algorithms could be even higher. However, if this holds in other settings needs to be investigated.

No major difference in the performance between both algorithms was found, where the simpler algorithm (algorithm 2) was only using microbiology culture data and no symptom information. One could have expected a bigger difference considering that during the ICU period there was only access to data on BSI symptoms in medical notes, and algorithm 1 needed structured symptom data, and if in general many cultures were taken regardless of symptoms and positive for contaminants. This might indicate that in this setting peripheral/central blood and CVC cultures were primarily taken in patients with symptoms of infection, in line with the culture recommendations in this setting and Swedish recommendations [32] which also matches the surveillance definition [27]. Consequently, this simpler algorithm could be used hospital-wide in this setting, independently from the availability of structured symptom data, without any major effect on the quality of surveillance. An advantage is that it is less vulnerable to weaknesses in data availability. Also, it could further facilitate the implementation in other health care systems since the demand for quality data on symptoms is removed. However, it is not certain that this algorithm without symptoms would perform equally well in other settings where clinical practices for taking blood cultures on wider indications are present.

We developed algorithms to detect all CVC-BSI cases and additionally to detect the ones that are HA according to ECDC’s criteria. The CVC-BSI cases that did not fulfil these HA criteria (> 30%) were long-term CVCs inserted during a previous hospital admission with a discharge more than 48 h before, e.g., a peripherally inserted central catheter (PICC-line) or subcutaneous venous port (SVP), or CVCs that were inserted at another healthcare facility than a hospital and still present when admitted to the hospital. Currently, patients get outpatient care with PICC-lines or SVPs or get it at another facility than the hospital [33]. Consequently, these cases should also be considered HA CVC-BSI even though not all are preventable for the hospital where they are admitted with their infection. Thus, the surveillance algorithm for all CVC-BSI cases better assesses their true burden on the healthcare system while the surveillance algorithm for HA CVC-BSI cases mostly indicates the cases possibly preventable by the hospital itself. Furthermore, the performance of the algorithms for HA cases was slightly lower than for all cases which is related to a larger misclassification of HA cases by the algorithms as they missed information that was only available in free-text.

Previous studies have demonstrated that it is possible to develop both semi- [13–16, 20–23] and fully-automated [17–19, 24, 25] surveillance algorithms for CVC-BSI according to the CLABSI definition that all perform well in comparison to manual record review. The heterogeneity in settings, populations and methods complicates a good comparison. Still, the performance of the algorithms developed in this study, for CVC-BSI according to the CRI3-CVC definition, are comparable to the results of these previous studies. Yet, as expected, the semi-automated algorithms in general had higher sensitivity and NPV combined with lower specificity and PPV compared to fully-automated algorithms which is in line with their different purpose and characteristics [6]. Based on the kappa analysis the agreement between the reference and the algorithm results in our study was mostly higher than in other studies [19–21] and similar to one other [24]. One study used the HELICS definition, the predecessor of ECDC’s definition, instead of CLABSI, but as this was a semi-automated algorithm their sensitivity was higher while their specificity and PPV was much lower than in our study [16]. Only two fully-automated algorithms were developed in a non-ICU or general hospital population [24, 25], using the CLABSI definition. The algorithm in the study of Herson et al. [25] had much lower sensitivity compared to our algorithms, but that algorithm was mainly based on diagnosis codes which is known to have poor sensitivity [34]. In the study of Woeltje et al. [24], their best performing algorithm, using culture, central line and fever data, achieved a higher sensitivity and lower specificity compared to the algorithms in this study. This difference might be explained by the different definitions used. CDC’s CLABSI definition [30] is a more non-specific definition compared to ECDC’s CRI3-CVC definition [27] and primarily works by excluding other sources of infection instead of linking it to the CVC, which is reflected in the algorithm rules used. Weaknesses in data quality, lack of documentation or reduced testing could more easily lead to more non-infected patients being classified as having an infection, both by manual review and algorithms, which consequently boosts the sensitivity for CLABSI-based algorithms. In contrast, infection is more difficult to ascertain for CRI3-CVC, both by manual review and algorithms, which could boost the specificity for these algorithms.

For the fully-automated algorithms CVC-BSI rates were either close to the true rates [24, 25] or overestimated [17, 19]. One can argue that for surveillance purposes it is not crucial whether the infection rate is over- or understated since it should primarily, as emphasised by Kaiser et al. [14], demonstrate changes in infection rates over time. However, an estimated rate close to the true rate would provide a better estimation of the true burden. In this study, the rates generated by both algorithms were close to the true rates. Due to high accuracy of the algorithms, they could possibly even be used for identifying individual patients for targeted interventions.

The rates of HA CVC-BSI by number of positive BCx or number of admissions in this study, 1.8% and 0.13%, is lower than what has been presented in the other studies which developed algorithms where these rates ranged from 6% till 36% and 0.64% till 6.2%, respectively [14–16, 19–22, 24]. Partly this might be explained by the fact that most rates were from the ICU instead of the total hospital population. However, also here the difference in definition of CVC-BSI used might play a role, as the CLABSI definition used in these studies in general gives higher rates of CVC-BSI than the CRI3-CVC definition used in this study [35, 36]. Usually, the incidence of CVC-BSI is expressed by catheter-days. As catheter-days could not reliable be obtained from structured data in our study, in-patient hospital-days were used as alternative. Our rate of HA-CVC-BSI of 0.20 per 1000 in-patient hospital-days is similar to the 0.20 per 1000 patient-days in a recent study in Spain which used the CRBSI definition which is fairly similar to the ECDC definition, but also included BSI related to peripheral venous catheters (PVC) [37].

The results of the second algorithm were plotted over a four-year period as a use-case for automated surveillance. It shows that it potentially could be used to indicate failure in infection prevention and control during strained healthcare situations like the COVID-19 pandemic. It also demonstrates the importance of showing both all CVC-BSI and HA CVC-BSI cases as, as mentioned above, one measures the overall burden and the other the part possibly preventable by the hospital itself. Subsequently, a change in the portion of HA CVC-BSI cases can indicate a change in the composition of the hospital population.

This study has several strengths. The study used a large dataset representative for the clinical population for which the algorithms are designed. Also, implementation in real life is facilitated as comprehensive EHR data was used. Furthermore, the algorithms have been validated on a separate dataset than the dataset used for development, in contrast to most of the previous studies, which increases the generalisability of the results [34, 38]. In addition, annotators were blinded to the algorithm results to prevent this from affecting their assessment while annotating the validation dataset. Finally, the international recognised and established criteria of ECDC and CDC were used for algorithm development and validation, and manual annotation was performed according to ECDC’s definition which increases the comparability and generalisability with other studies. However, some limitations should also be considered. The algorithms have not been validated in the obstetric population. However, hardly any positive BCx were present during obstetric admissions, so this would probably not greatly influence the results. The developed algorithms focus only on microbiologically confirmed CVC-BSI based on the CRI3-CVC definition and not on local, general, or non-microbiology confirmed CVC-related infection (CRI1-CVC, CRI2-CVC, or C-CVC) nor on PVC-related infections which are also important infections. Consequently, the true burden of CVC-BSI is probably underestimated, and the burden of all CRI is not measured. Even though a different dataset was used for the development and validation, they were coming from a similar population and therefore, the algorithms still need to be validated in other settings and EHR systems to better assess their generalisability. As in this study setting, neither the semi-quantitative method for CVC cultures nor the quantitative blood culture ratio between central and peripheral blood samples is determined, these criteria were not incorporated in the algorithms and need to be added and validated in settings where these methods are used. Although comprehensive EHR data was used, implementation and evaluation in real life settings is still needed. While these developed fully-automated surveillance algorithms could replace manual surveillance, it remains important to consider that due to changes in methods and reporting over time they still need regular readjustment and revalidation. It is likely that the CVC-BSI rates, by both manual review and the algorithms, underestimated the ‘true’ rate as patients are possibly not cultured appropriately (e.g., no BCx taken while symptoms are present). Finally, wrong or missing information in the EHR system could have influenced algorithm performance, but this affects manual surveillance in a similar fashion.

## Conclusions

The present study indicates that it is possible to develop an algorithm based on ECDC’s definition of CVC-BSI (CRI3-CVC) that is appropriate for continuous automated surveillance purposes and performs well in comparison to manual record review and previously developed algorithms using CDC’s CLABSI criteria. The simpler algorithm, only using microbiology data, can be used in settings with testing for CVC-BSI adhering to recommendations. However, in other settings the algorithm including symptom data might need to be applied and further validation of both algorithms in other settings is needed to assess the generalisability of the algorithms.

## Data Availability

The datasets generated and/or analysed during the current study are not publicly available due to ethical limitations related to sharing patient information, but are available from the corresponding author on reasonable request.

## Abbreviations

AUROC: Area under the receiver operating characteristic curve
BCx: Blood culture
BSI: Bloodstream infection
CDC: Centers for Disease Control and Prevention
CFU: Colony-forming unit
CI: Confidence interval
CLABSI: Central line-associated bloodstream infection
CRI: Catheter-related infection
CRI3-CVC: Microbiologically confirmed central venous catheter-related bloodstream infection
CVC: Central venous catheter
CVC-BSI: Central venous catheter-related bloodstream infection
ECDC: European Centre for Disease Prevention and Control
EHR: Electronic health record
HA: Healthcare-associated
HAI: Healthcare-associated infection
ICU: Intensive care unit
IQR: Interquartile range
KUH: Karolinska University Hospital
NPV: Negative predictive value
PICC: Peripherally inserted central catheter
PPV: Positive predictive value
RIT: Repeat infection timeframe
SVP: Subcutaneous venous port
